# Characteristics of Organic Acid Secretion Associated with the Interaction between* Burkholderia multivorans* WS-FJ9 and Poplar Root System

**DOI:** 10.1155/2018/9619724

**Published:** 2018-12-31

**Authors:** Guan-Xi Li, Xiao-Qin Wu, Jian-Ren Ye, He-Chuan Yang

**Affiliations:** ^1^Co-Innovation Center for Sustainable Forestry in Southern China, College of Forestry, Nanjing Forestry University, Nanjing, Jiangsu 210037, China; ^2^Academy of Agricultural Sciences of Lianyungang, Lianyungang, Jiangsu 222006, China; ^3^School of Life Science, Qufu Normal University, Qufu, Shandong 273165, China

## Abstract

The objective of this study was to investigate whether plant-bacteria interaction affects the secretion of organic acids by both organisms and to assess whether the production of IAA by the bacterium increases the secretion of organic acids by root exudates, and if the stress produced by low available phosphorus (P) affects the production of organic acids by bacteria, by roots, or by root exudates in presence of bacterial cultures. With this purpose, we used as a biological model poplar plants and one strain of* Burkholderia multivorans* able to solubilize P. High performance liquid chromatography was utilized to measure organic acids. The tests, the inductive effects of exogenous indole-3-acetic acid (IAA) on secretion of organic acids, the 2 × 4 × 2 factorial design experiment, and the ability of organic acids to solubilize tricalcium phosphate were performed to investigate the interactive effects. The results showed that, after* B. multivorans* WS-FJ9 interacted with the poplar root system, the key phosphate-solubilizing driving force was gluconic acid (GA) which was produced in three ways: (1) secreted by the root system in the presence of IAA produced by* B. multivorans* WS-FJ9; (2) secreted by* B. multivorans* WS-FJ9; and (3) secreted by the poplar root system in the presence of phosphorus stress. When phosphorus stress was absent, the GA was produced as outlined in (1) and (2) above. These results demonstrated that inoculating* B. multivorans* WS-FJ9 into the poplar root system could increase the amount of GA secretion and implied that the interaction between* B. multivorans* WS-FJ9 and the poplar root system could contribute to the increase of P available fraction for poplar plants.

## 1. Introduction

Interaction between microorganisms and plants refers to the process in which microorganisms affect plant growth through their own metabolism and plants also have an influence on microbial community composition and quantity. Plant growth promoting rhizobacteria (PGPR), which widely exist in many kinds of plant rhizospheres, is a kind of beneficial bacteria that can promote plant growth and inhibit harmful microbes [1–5]. The interactions between some PGPR and plants can enhance the secretion of organic acids [6, 7], which play an important role in the process of the activation and absorption of insoluble nutrients by plant [7–9]. Phosphate-solubilizing bacteria (PSB)—which are able to secrete organic acids, hormones, and some active substances—are also PGPR [10–12].

Organic acids secreted by solubilizing bacteria or by plants include oxalic, tartaric, acetic, citric, butyric, propionic, malonic, lactic, succinic, malic, acetic, fumaric and adipic acids and gluconic acid (GA), and keto-GA [13–19] and play important roles in the phosphate-solubilizing process. The type and amount of organic acids secreted by different PSB and plant are variable [20–22], and different organic acids play various roles in the phosphate-solubilizing process. Ahuja suggested that citric and oxalic acids produced by* Paecilomyces marquandii *AA1 were essential factors for solubilizing phosphate [23]. Rodriguez and Hameeda proposed that the ability of microorganisms to solubilize inorganic phosphate was positively correlated with the amount of GA produced [24]. Liu reported that oxalic and tartaric acids secreted by* Pseudomonas fluorescens* JW-JS1 played an important role in the process of solubilizing tricalcium phosphate [Ca_3_(PO_4_)_2_] [25]. Plant roots can produce a certain amount of organic acids, which enables plant roots to have a certain ability to solve inorganic phosphorus. Hormones can induce plant roots to secrete organic acids [26–29], indicating that hormones can promote the solubilizing insoluble phosphorus and uptake of available phosphorus by plant roots.

*Burkholderia multivorans* WS-FJ9, obtained in our laboratory by screening from* Pinus elliottii* rhizosphere, is a highly-effective phosphate-solubilizing strain. Preceding studies have shown that this bacterial strain had an excellent ability to solubilize inorganic phosphate with a significant effect on promoting poplar growth and was able to colonize the poplar rhizosphere [30–33]. However, the mechanism and pathways of solubilizing inorganic phosphate by this strain are still unidentified. In this study, by using liquid culture technique and high performance liquid chromatography (HPLC), we explored the characteristics of secreting organic acids and indole-3-acetic acid (IAA) by strain WS-FJ9 and the poplar root system, effects of exogenous IAA on secretion of organic acids by* B. multivorans* WS-FJ9 and the poplar root system, and effects of the rhizospheric interaction between* B. multivorans* WS-FJ9 and the poplar root system on secretion of organic acids and investigated the phosphate-solubilizing mechanism based on the interaction between microorganisms and plants.

## 2. Materials and Methods

### 2.1. Experimental Materials

#### 2.1.1. Experimental Strain and Preparation of Bacterial Suspension

*Burkholderia multivorans *WS-FJ9, screened by our laboratory and later preserved at the China Center for Type Culture Collection (CCTCCM2011435) [31], was used. After activation, a single colony of* B. multivorans* WS-FJ9 was picked with an inoculating loop and inoculated into a 100-mL flask containing 50 mL of nutrient broth (tryptone 10 g, beef extract 3 g, NaCl 5 g, distilled water 1000 mL, and pH7.4), followed by incubation on a shaker at 28°C with rotation speed of 200 r·min^–1^ for 48 h to obtain seed bacteria. And then 0.5 mL of seed bacteria was transferred into a 100-mL flask containing 50 ml of nutrient solution by pipette to cultivate under the same culture conditions, and this process was repeated to get more bacterial suspensions. Finally, the bacterial suspension was centrifuged at 7000 r·min^–1^and 4°C, for 5 min to collect bacterial precipitate, which was then rinsed three times adjusted to 1×10^8^ cfu·mL^–1^ using sterile saline solution.

#### 2.1.2. Experimental Plants

Poplar “NL-895” (*Populus *×* euramericana *cv.) was used [34]. Seeds of poplar NL-895 were first immersed in 0.1% KMnO_4_ for 2 h, followed by rinsing them three times with sterile water. Then, the seeds were sown in sterilized river sand. At 40 d after emergence, poplar seedlings of similar growth were transplanted into pots each containing 1.5 kg of an autoclaved mixture of paddy soil and peat moss at 2:1 (v/v), in which available nitrogen, phosphorus, and potassium were 30.0, 6.0, and 23.5 mg·kg^–1^, respectively. Next, all seedlings were relocated into a greenhouse (Intensity of Illumination 20000 Lux, Temperature 25°C) and watered once every two days, and under the same management for all seedlings. One year later, the seedlings together with pots were first immersed into Hoagland nutrient solution for 10 d. Afterwards, the pots were removed and soil adhering to seedling roots was washed off, while avoiding damage to roots. Then, the poplar seedlings were submerged in distilled water for 3 h, followed by cultivation in modified Hoagland nutrient solution (see the followed section for the description of this medium) which was replaced at 5-d intervals. After 20 d, the seedlings were removed from the nutrient solution and immersed in distilled water for 3 h. Then the seedlings having the same growth status and biomass were chosen and cultivated in 600-mL light-blocking glass bottles, respectively, containing 0-, low-, normal-, and high-phosphate nutrient solutions (see the following section “Experimental Nutrient Solution”), with one seedling per bottle. The secretion of organic acids by plants is affected by plant status, such as shoot and root growth, growth stage, and nutrient status. Therefore, all experiments in this study used poplar seedlings of similar growth status and biomass.

#### 2.1.3. Experimental Nutrient Solution

The experimental nutrient solutions were shown in Table 1. These nutrient solutions were transferred into glass bottles, in which 0.2 mg of Ca_3_(PO_4_)_2_ (Aladdin Industrial Corporation, Shanghai, China. AR, ≥96.0%) per mL of nutrient solution was added.

#### 2.1.4. Chromatographic Conditions for Organic Acid Measurement

HPLC was used to measure the content of organic acids with an Agilent 1200 liquid chromatograph and Thermo Hypersil Gold chromatographic columns (5 *μ*m, 4.6 × 250 mm). The chromatographic parameters were measuring wavelength of 203 nm, column temperature at 25°C, 50 mmol·L^–1^ KH_2_PO_4_ (pH 2.5) (A) and CH_3_CN (B) as mobile phase, volume of loaded sample of 2 *μ*L, flow rate of 1.0 mL·min^–1^, and gradient: 100%A (0–8 min), A/B (3:2) (8–13 min).

### 2.2. Experimental Methods

#### 2.2.1. Measurement of IAA Content in* B. multivorans* WS-FJ9 Medium and Poplar Nutrient Solution

IAA content was measured by the method of Bric* et al.* with slight modifications [35]. Tryptophan solution of 2.5 mg·mL^–1^ was prepared and then filter sterilized. Subsequently, it was added into a 100-mL flask containing 50 mL of Tryptone Soya Broth medium autoclaved at 121°C for 20 min and the final tryptophan concentration was adjusted to 0.5 mg·mL^–1^. Afterwards, 100 *μ*L of* B. multivorans* WS-FJ9 seed (1×10^8^ cfu·mL^–1^) solution was inoculated into each flask, followed by incubation on a shaker at 28°C and 200 r·min^–1^. The culture was sampled every 24 h for measurement of optical density value at 630 nm (*OD*_630_) as the bacterial growth index. Meanwhile, the fermentation broth was centrifuged at 25°C and 6000 r·min^–1^ for 10 min to collect the supernatant. Tryptophan solution was also added into the nutrient solution of poplar seedlings at a final concentration of 0.5 mg·mL^–1^, followed by filtering with a 0.22-*μ*m microfiltration membrane. Afterwards, 50 *μ*L of 10 mM orthophosphoric acid and 2 mL of Sakowski chromogenic agent (7.5 mL of 0.5 M FeCl_3_·6H_2_O, 150 mL of 18 M sulfuric acid and 250 mL of deionized water) were added into 1 mL of the supernatant of* B. multivorans* WS-FJ9 fermentation broth and 1 mL of the filtered nutrient solution of poplar seedlings, respectively, followed by incubation in darkness for 30 min at 25°C. Subsequently, the absorbance of each reaction solution was measured at 530 nm. Distilled water was used as the control for zero adjustment and the standard curve was drawn with IAA (Sigma-Aldrich Co. LLC., St Louis, USA, Purity 98%) standard solutions at 0, 5, 10, 20, 30, 40, 50, and 60 mg·L^–1^ to determine the IAA concentration in the* B. multivorans* WS-FJ9 fermentation broth and the nutrient solution of poplar seedlings.

#### 2.2.2. Measurement of Organic Acid Content in the* B. multivorans* WS-FJ9 Fermentation Broth and the Nutrient Solution of Poplar Seedlings

In total, 100 *μ*L of* B. multivorans* WS-FJ9 seed solution was inoculated into a 100-mL flask containing 50 mL of NBRIP (the phosphatic growth medium of the International Plant Nutrition Institute, Norcross, GA, USA) [36], followed by incubation on a shaker at 28°C and 200 r·min^–1^ for 72 h. Afterwards, the fermentation broth was centrifuged at 4°C and 10 000 r·min^–1^ for 10 min. The obtained supernatant was first filtered with a 0.22-*μ*m microfiltration membrane and then loaded in chromatographic columns to measure the organic acid content by HPLC. NBRIP medium without inoculation of* B. multivorans* WS-FJ9 was used as a blank control.

The normal-P nutrient solution of poplar seedlings cultured was first filtered with a 0.22-*μ*m microfiltration membrane and then concentrated by rotation at 40°C to 15 mL, followed by adding 1.5 mL of 20 mM Na-EDTA to eliminate the effect of metal ions on organic acids in the concentrated solution [37]. Finally, the volume was adjusted to 20 mL and then loaded in chromatographic columns for measurement of organic acid content.

#### 2.2.3. Induction of Organic Acid Secretion by Exogenous IAA in the Poplar Root System and* B. multivorans* WS-FJ9

For investigating the effects of exogenous IAA on organic acids secreting by WS-FJ9, IAA was added into 100-mL flasks each containing 50 mL of NBRIP to obtain respective final concentrations of 0, 5, 10, 20, 30, and 40 mg·L^–1^. Then, to each was added 100 *μ*L of* B. multivorans* WS-FJ9 bacterial suspension, followed by shaking culture at 28°C with rotation speed of 200 r·min^–1^ for 3 d. Subsequently, GA and shikimic acid contents in the fermentation broth were measured.

For exploring the effects of different concentrations of exogenous IAA on organic acids secreting by poplar roots, poplar seedlings were first transferred into glass culture bottles each containing 200 mL of 0-phosphorus nutrient solution. Then, IAA was added to each bottle to set an IAA concentration gradient of 0, 5, 10, 20, 30, and 40 mg·L^–1^ in corresponding treatments, with three replicates each. The seedlings were cultivated in a greenhouse, followed by measurement of organic acid content in the nutrient solutions 24 h later.

In order to investigate the effects of exogenous IAA on organic acids secreting by the poplar roots which were in different phosphate levels of the nutrient solutions, poplar seedlings were first transferred into glass culture bottles, respectively, containing 200 mL of 0-, low-, normal-, and high-phosphate nutrient solutions. Next, IAA was added into each bottle at a final concentration of 20 mg·L^–1^, with three replicates per treatment. The seedlings were cultivated in a greenhouse, followed by measurement of organic acid content in the nutrient solutions 24 h later.

#### 2.2.4. Interaction between* B. multivorans* WS-FJ9 and the Poplar Root System

First, glass bottles, respectively, containing 200 mL of 0-, low-, normal-, and high-phosphorus nutrient solutions were prepared (See “Experimental Nutrient Solution” for more details). Then, poplar seedlings were transferred into a batch of these bottles containing nutrient solutions of four different phosphate levels, followed by addition of 50 mL of* B. multivorans* WS-FJ9 suspension (1 × 10^8^ cfu·mL^–1^). Additionally, in one control treatment, poplar seedlings were transferred into another batch of glass bottles containing nutrient solutions of four different phosphate levels, followed by adding of 50 mL of sterile water; in the other control treatment, only 50 mL of* B. multivorans* WS-FJ9 suspension (1 × 10^8^ cfu·mL^–1^) was added into a batch of glass bottles containing nutrient solutions with four different phosphate levels. An experiment with a 2 × 4 × 2 factorial design was used to evaluate the effect of the interaction between WS-FJ9 and the poplar root system on GA secretion, with three replicates per treatment. After cultivation for 24 h in a greenhouse, the contents of organic acids and IAA in the nutrient solutions were measured.

#### 2.2.5. Phosphate-Solubilizing Test of Exogenous Organic Acids

With reference to the result of measurement of organic acids secreted by the poplar root system exposed to* B. multivorans* WS-FJ9 and phosphorus stress, aqueous solutions of GA and oxalic, shikimic, lactic, and malonic acids (these chemicals were purchased from Aladdin Industrial Corporation, Shanghai, China, gluconic acid: 49-53 wt. % in H_2_O; oxalic acid: AR, ≥99.5%; shikimic acid: ≥98%%; lactic acid: AR, ≥90%; malonic acid: AR, ≥99.5%) were prepared with distilled water, and the final concentration and pH value were shown in Table 2. Then, 50 mL of each aqueous solution was, respectively, pipetted into each 100-mL flask, followed by addition of 5 g of Ca_3_(PO_4_)_2_ (Aladdin Industrial Corporation, Shanghai, China, AR, ≥96.0%). Flasks containing 50 mL of distilled water with 5 g of Ca_3_(PO_4_)_2_ added were used as blank controls. After autoclaving at 121°C for 20 min, flasks were first incubated on a shaker at 28°C and 200 r·min^–1^ for 72 h, and then the solution was centrifuged at 10 000 r·min^–1^ at 4°C for 10 min. The content of dissolved phosphorus in supernatant was measured using Mo-Sb colorimetry [38]. The experiment was repeated twice.

#### 2.2.6. Data Analysis and Processing

Software Origin 8.6 (Origin Lab Corporation, Northampton, MA, USA) was used for statistical analysis, linear fitting, significance testing (*P *< 0.05), and charting of IAA secretion in WS-FJ9 and growth dynamic of bacterial cells, effect of exogenous IAA on secretion of organic acids by* B. multivorans* WS-FJ9 and on secretion of organic acids by the poplar root system, and effects of different organic acids on solubilization of Ca_3_(PO_4_)_2_. SPSS 16.0 (SPSS Inc., Chicago, USA) and SAS 9.1 (SAS Institute Inc., North Carolina, USA) software were used to analyze the 2 × 4 × 2 factorial experiment for interaction of* B. multivorans* WS-FJ9 and the poplar root system.

## 3. Results

### 3.1. Determination Results of IAA Content in* B. multivorans* WS-FJ9 Medium and Poplar Nutrient Solution

**Figure 1:**
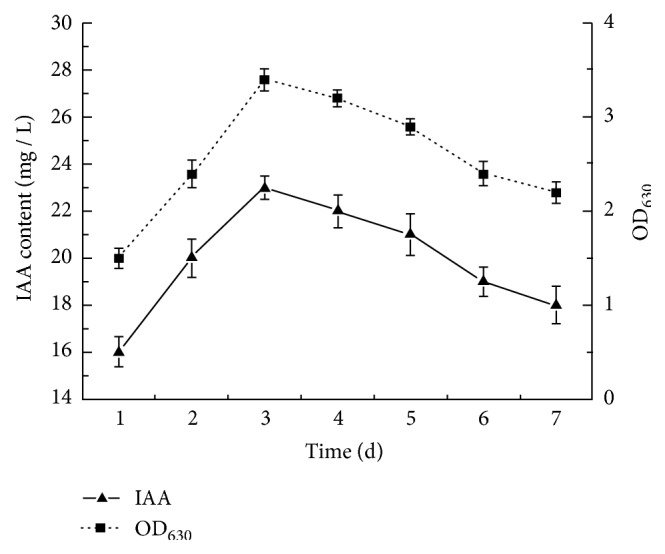
**IAA secretion in strain WS-FJ9 and growth dynamic of bacterial cells.** *OD*_630_ represents the proliferation of bacteria. At the initial stage of incubation (1–3 d), both the IAA content and bacterial biomass (denoted by* OD*_630_ value) in the fermentation broth of* B. multivorans* WS-FJ9 increased quickly and peaked at 3 d, with values of 23 mg·L^–1^ and 3.4, respectively.

IAA was detected in the bacterial medium and not in the poplar nutrient solution. IAA secretion by* B. multivorans *WS-FJ9 and growth dynamic of bacterial cells were that at the initial stage of incubation (1–3 d), both the IAA content and bacterial biomass (denoted by* OD*_630_ value) in the fermentation broth of* B. multivorans* WS-FJ9 increased quickly and peaked at 3 d, with values of 23 mg·L^–1^ and 3.4, respectively (Figure 1). Afterwards, both indices showed decreasing trends, with corresponding values of 18 mg·L^–1^ and 2.2 at 7 d. The results showed that the amount of bacteria affected the secretion of IAA. After inoculation, the growth of bacteria entered the logarithmic phase after one day, and the number of bacteria and the secretion of IAA increased rapidly; after 3 days, entered stationary phase, the number of bacteria and the secretion of IAA reached a maximum; subsequently, entered decline phase, the number of bacteria and the secretion of IAA began to decrease. So the growth stage of bacteria can play a pivotal role in the production of IAA.

### 3.2. Types of Organic Acids in the Fermentation Broth of* B. multivorans* WS-FJ9 and Effect of Exogenous IAA on Secretion of Organic Acids by* B. multivorans* WS-FJ9

**Figure 2:**
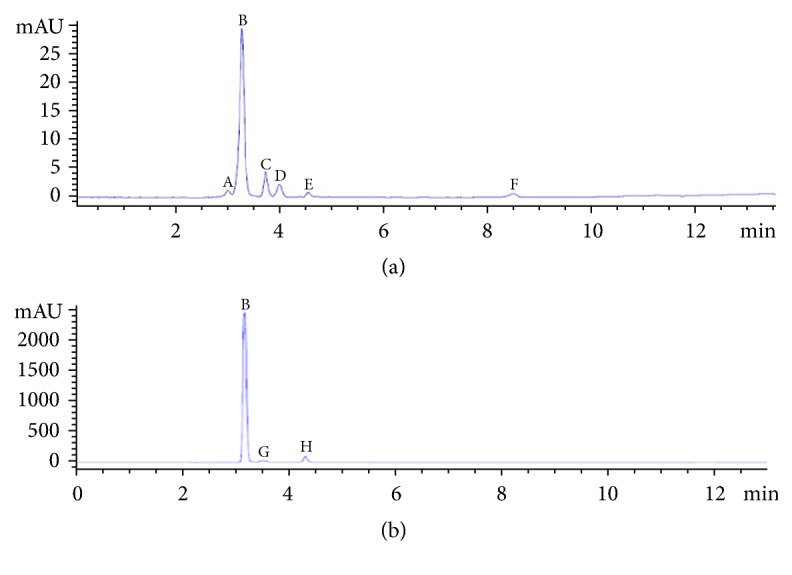
**HPLC chromatogram of the strain WS-FJ9 fermentation broth and the poplar culture solution**. (a) The strain WS-FJ9 fermentation broth; (b) the poplar culture solution; (A) oxalic acid; (B) gluconic acid; (C) shikimic acid; (D) lactic acid; ((E), (F), and (G)) unknown acid; (H) malonic acid.

Chromatography analysis showed that organic acids including GA and shikimic, lactic, oxalic, and some unknown acids were present in the fermentation broth of* B. multivorans* WS-FJ9 after 3 d of incubation (Figure 2(a)). Among them, the content of GA was the highest (667.35 mg·L^–1^) and those of shikimic, lactic, and oxalic acids were 136.85, 87.48, and 14.06 mg·L^–1^, respectively. The amount of GA was significantly higher than for the other acids secreted, implying that GA played a more important role in rhizosphere ecology and possibly phosphate solubilization.

**Figure 3:**
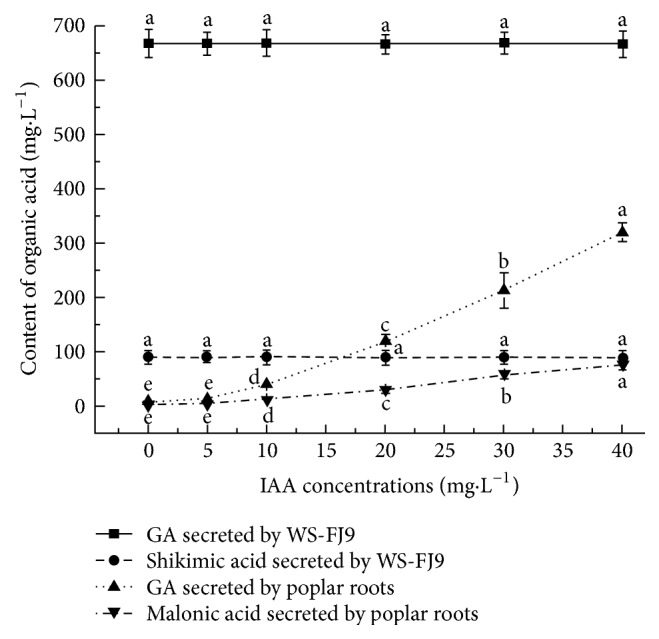
**Induced effect of different concentrations of IAA on the secretion of organic acids.** *B multivorans* and poplar were cultured separately. The contents of both GA and shikimic acid among the fermentation broths of* B. multivorans* WS-FJ9 with different IAA concentrations added had no significant differences, and the contents of both GA and malonic acid in 0-phosphorus nutrient solution cultured poplar were increased with IAA concentration.

ANOVA showed no significant differences in contents of GA and shikimic acid among the fermentation broths of* B. multivorans* WS-FJ9 with different IAA concentrations added (Figure 3). This indicated that the secretions of GA and shikimic acid in the fermentation broths of* B. multivorans* WS-FJ9 are not affected by IAA.

### 3.3. Types of Organic Acids Secreted by the Poplar Root System and Effect of Exogenous IAA on Their Secretion

Chromatography analysis showed that GA and malonic and an unknown acid were detected after poplar seedlings were cultivated in 0-phosphorus nutrient solution for 24 h (Figure 2(b)), and the contents of GA and malonic acid were 122.35 and 14.75 mg·L^–1^, respectively, compared to corresponding values of only 17.95 and 2.46 mg·L^–1^, in controls with normal phosphorus level. The much higher content of organic acids in the treatment of phosphorus stress revealed that the poplar root system could relatively secrete more GA and malonic acid under this condition and thus was capable of solubilizing inorganic phosphate to some degree.

In the present study, when different concentrations of IAA were added to 0-phosphorus nutrient solution to cultivate poplar seedlings, after 24 h, the contents of both GA and malonic acid in the solution increased with IAA concentration (Figure 3). Taking the content of organic acid as the dependent variable (*Y*) and IAA concentration as the independent variable for linear regression analysis, the contents of GA and malonic acid showed very high positive correlations with IAA concentration within the concentration range of 5–40 mg·L^–1^, with the obtained linear equations:* Y* = 8.8365*X* – 44.835 (*R*^2^ = 0.9912,* P *< 0.01) and* Y* = 2.0658*X* – 7.7584 (*R*^2^ = 0.9934,* P *< 0.01), respectively.

**Figure 4:**
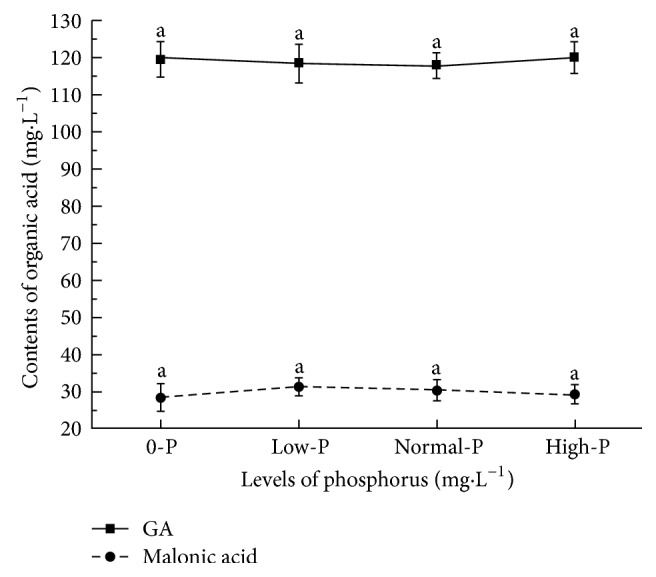
**Induced effect of 20 mg·L** ^–1^ **IAA on poplar roots secreting organic acids.** The contents of both GA and malonic acid in different nutrient solutions cultured poplar had no significant differences. The normal-P solution was the modified Hoagland nutrient solution containing 136 mg·L^–1^ KH_2_PO_4_ and for the 0-P nutrient solution, KH_2_PO_4_ was replaced by 74.55 g of KCl and pH value was 6.8; for the low-P solution, every 68 g KH_2_PO_4_ was replaced by 37.3 g of KCl and pH value was 6.7; and for the high-P solution, the content of KH_2_PO_4_ was doubled compared to the normal-P solution and pH value was 6.4.

ANOVA showed no significant differences in the contents of GA and malonic acid among 0-, low-, normal-, and high-phosphorus poplar nutrient solutions containing 20 mg·L^–1^ IAA (Figure 4). Induction of the secretion of organic acids in the poplar root system by IAA was not affected by phosphate content in the environment. This implied that there was no correlation between the induction of acid secretion by IAA in root system and the induction of acid secretion by phosphorous stress in the root system. This may be because the mechanisms for IAA and phosphorus stress inducing the root system to produce acids differ. Thus, the two pathways were independent of each other.

### 3.4. Effect of Interaction between* B. multivorans* WS-FJ9 and the Poplar Root System on Secretion of Organic Acids

**Figure 5:**
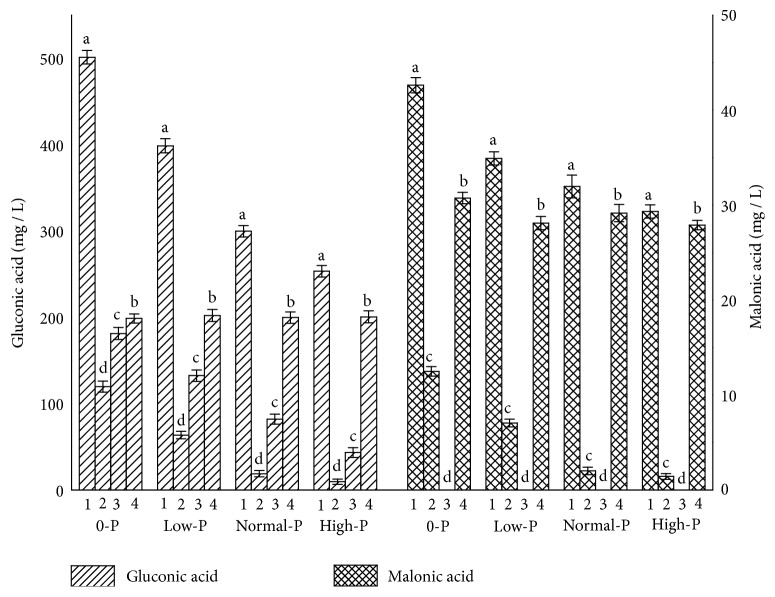
**Effect of rhizospheric interaction between strain WS-FJ9 and the poplar root system.** 1: organic acid secreted with the interaction; 2: organic acid secreted by the poplar roots without IAA-induced; 3: organic acid secreted by WS-FJ9; 4: organic acid secreted by the poplar roots with IAA-induced.

The contents of GA and malonic acid measured in nutrient solutions in the present study are shown in Figure 5. Those induced by IAA secreted by WS-FJ9 in nutrient solutions were calculated using the regression equations* Y* = 8.8365*X* – 44.835 and* Y* = 2.0658*X* – 7.7584. GA was the major organic acid secreted with the interaction between the poplar root system and* B. multivorans* WS-FJ9. Under phosphorus stress conditions, the organic acids secreted came from three main sources/factors: IAA-induced secretion in the poplar root system, secretion by* B. multivorans* WS-FJ9, and phosphorus stress-induced secretion in the poplar root system. Among these, the content of organic acids from the latter two showed decreasing trends with increased phosphorus content in nutrient solution and that from the first source was basically the same in nutrient solutions with different phosphorus levels, while, under 0-phosphorus stress condition, organic acids secreted under the interaction between the poplar root system and* B. multivorans* WS-FJ9 were mainly secreted by the poplar root system induced by IAA and by* B. multivorans* WS-FJ9. This indicated that GA secretion plays an important role for* B. multivorans* WS-FJ9 to solubilize inorganic phosphate, consistent with the conclusion of Hameeda [24], and suggested that secretion of organic acids induced by* B. multivorans* WS-FJ9 in the poplar root system was the major mechanism of phosphate solubilization.

**Figure 6:**
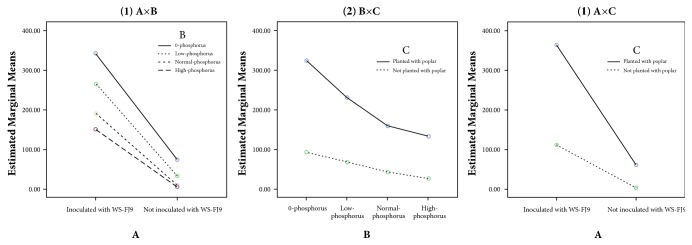
**Estimated marginal means of GA.** (A) Inoculation of* B. multivorans* WS-FJ9; (B) phosphorus content of the nutrient solutions; (C) poplar root system. Two-way interactions between any two factors on GA production were significant (*P *< 0.05).

Tests of Between-Subjects Effects and multiple comparisons of the 2 × 4 × 2 factorial design experiment were performed (Tables 3 and 4). WS-FJ9 (factor A), P content (factor B), and the poplar root system (factor C) significantly affected GA production (*P *< 0.05). Two-way interactions between any two factors on GA production were significant (*P *< 0.05). However, the three-way interaction was not significant. As shown in Figure 6, all of the factor lines in (1), (2), and (3) were not paralleled and gradually close to each other, and the factor lines of high-phosphorus and not planted with poplar were in the lower. It implied that inoculating* B. multivorans* WS-FJ9 and phosphorus stress could promote GA secretion and that poplar roots were able to secrete GA under phosphorus stress. As shown in Table 2, inoculating* B. multivorans* WS-FJ9 and planting poplar could promote GA secretion. Treatment B1 (0-phosphorus) had significantly higher GA secretion than other phosphorus levels (*P *< 0.05) and indicated that phosphorus stress could promote GA secretion. These analyses demonstrated that inoculating* B. multivorans* WS-FJ9 into the poplar root system under phosphorus stress was able to make the amount of GA secretion highest and implied that* B. multivorans* WS-FJ9 and the poplar root system had strong capacity for solubilizing phosphate under phosphorus stress. When* B. multivorans* WS-FJ9 interacted with the poplar root system, GA was the key phosphate-solubilizing driving force and was produced in three ways: (1) secreted by the root system in the presence of IAA produced by* B. multivorans* WS-FJ9; (2) secreted by* B. multivorans* WS-FJ9; and (3) secreted by the root system in the presence of phosphorus stress. In the absence of phosphorus stress, GA was produced as outlined in (1) and (2) above.

### 3.5. Effects of Different Organic Acids on Ca_3_(*PO*_4_)_2_ Solubilization

**Figure 7:**
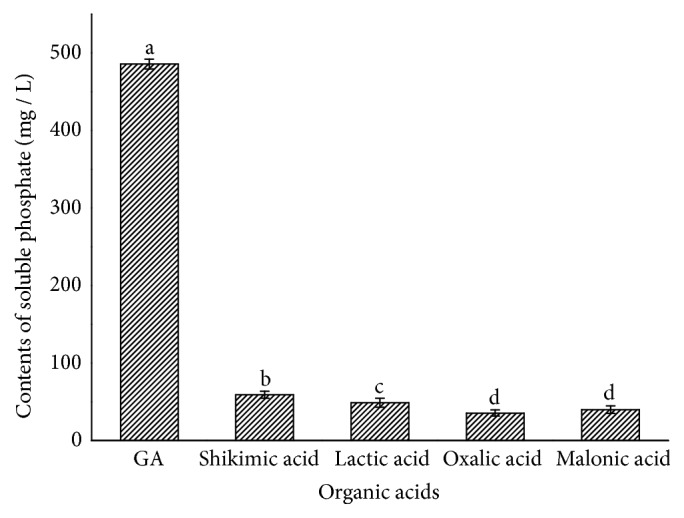
**The effect of different organic acids on solubilization of** **C** **a**_3_(**PO**_4_)_2_. The amount of soluble phosphate solubilized by GA was significantly higher than by other organic acids.

The five kinds of organic acids found could solubilize Ca_3_(PO_4_)_2_, most notably GA with the amount of solubilized phosphate of 485.65 mg·L^–1^, 2.6 times the total amount of phosphate solubilized by other organic acids (Figure 7). This indicated that GA secreted by WS-FJ9 was the key acid for solubilization of inorganic phosphate. Our results showed that GA produced by the interaction between the poplar root system and* B. multivorans* WS-FJ9 came from three sources, most secreted by the root system induced by IAA generated by* B. multivorans* WS-FJ9, and least secreted by the root system without IAA induction. Therefore, under both phosphorus stress and 0-phosphorus stress conditions, GA secreted by the root system induced by IAA produced by* B. multivorans* WS-FJ9, and that secreted by* B. multivorans* WS-FJ9, and that secreted by root system induced by phosphorus stress were the major causes of phosphate solubilization. This also revealed that the poplar root system itself possessed the ability to solubilize phosphate to some extent.

## 4. Discussion

Phosphate-solubilizing microorganisms can secrete a variety of organic acids and achieve the degradation of insoluble phosphate through acidolysis or complexation [39]. Louw and Webly proved that even when bacteria were inactivated, organic acids could still solubilize phosphate, and their effect depended on their types and the amount secreted [40]. Hameeda* et al.* showed that the phosphate-solubilizing ability of microorganisms was related to the amount of GA produced—with the more GA secreted, the higher the phosphate-solubilizing ability [24]. In our study, several kinds of organic acids were detected in the fermentation broth of strain WS-FJ9, of which GA was the highest (the content reached 667.35 mg · L^−1^, much higher than the secretion of other organic acids). Meanwhile, subsequent tests also indicated that GA had the best effect of solubilizing insoluble P sources (this was shown in the experiment of “phosphate-solubilizing test of exogenous organic acids”). This indicated that the secretion of GA plays an important role for strain WS-FJ9 to solubilize inorganic phosphate, which is consistent with the conclusion drawn by Hameeda* et al. *[24]. And we also studied the organic acids secreted by poplar root system, from which gluconic acid, malonic acid, and an unknown acid were detected in the secreta of poplar root system under phosphorus stress condition, and the contents of malonic acid and glucose were 122.35 mg · L^−1^ and 14.75 mg · L^−1^, extremely significantly higher than their counterparts under the condition with normal phosphorus level, indicating that poplar root system can relatively secrete more glucose acid and malonic acid under phosphorus stress condition thus capable of solubilizing inorganic phosphate to a certain degree.

Hormones can induce plant roots to secrete organic acids, possibly because they can act as signal substances to induce the opening of organic acid anion channels in the root system. IAA is involved in the regulation of anion channels and plays a role in determining the opening and closing of organic acid anion channels [41, 42]. Previous studies have shown that abscisic acid (ABA) can regulate the secretion of oxalic acid in buckwheat and significantly induce secretion of citric acid at the root tips of soybean [26, 27]. IAA can induce secretion of malic acid in oat and wheat [28, 29]. In our study, effects of exogenous IAA at different concentrations on the secretion of organic acids by poplar root system and strain WS-FJ9 and effect of IAA at the same concentration on the secretion of organic acids by poplar root system in poplar nutrient solutions with different phosphorus levels were also investigated, from which it was shown that both the contents of malonic acid and gluconic acid in poplar nutrient solution revealed extremely significant linear correlation with the amount of added IAA within IAA concentration range of 5-40 mg·L^−1^ under phosphorus stress condition; while the amount of added IAA had no effect on the secretion of organic acids in strain WS-FJ9, induction of acid secretion by IAA in poplar root system was not affected by phosphorus content in environment; that is, there was no correlation between the induction of acid secretion by IAA in root system and the induction of acid secretion by phosphorous stress in root system, which may be due to the fact that the mechanisms for IAA and phosphorus stress to induce root system to produce acids are different; thus the two pathways are independent with interference from other. This indicated that IAA can induce the generation of a certain ability of poplar roots to solve inorganic phosphorus. Meanwhile, we measured the secretion of IAA in* B. multivorans* WS-FJ9 medium and poplar nutrient solution. The results showed that IAA was detected in the bacterial medium and not in the poplar nutrient solution. And the content of detected IAA reached up to 23 mg·L^−1^ in the fermentation broth of strain WS-FJ9 after incubation for three days. It was speculated that the IAA secreted by this strain could induce the generation of a certain ability of poplar roots to solve inorganic phosphorus.

Mechanisms of phosphate solubilization in phosphate-solubilizing bacteria are complex and diverse. In addition to the opinion that the insoluble phosphate is solubilized through acidolysis by the secreted organic acids, Asea has proposed that certain microorganisms solubilize the insoluble inorganic phosphate through respiration or NH_4_^+^ assimilation to produce protons other than generation of organic acids [43], while Narsian and Patel have presented that the generation of proton is not directly related to amount of phosphorus dissolved by phosphate-solubilizing microorganisms [17]; Yi has pointed out that polysaccharides secreted by microorganisms play an important role in the microbial phosphate-solubilizing process [44]. All the opinions above concerning phosphate-solubilizing mechanism in microorganisms are presented based on the microbial strains used in their individual studies; thus there remains no common understanding on this issue [34]. Our study has showed that gluconic acid is the major organic acid produced under the interaction between strain WS-FJ9 and plant root system; gluconic acid secreted by root system induced by IAA produced by strain WS-FJ9 and secreted by strain WS-FJ9 was the major phosphate-solubilizing power under nonphosphorus stress, while, under phosphorus stress condition, gluconic acid secreted by root system induced by IAA produced by strain WS-FJ9, and that secreted by strain WS-FJ9, and that secreted by poplar root system induced by phosphorus stress were the major phosphate-solubilizing power. Phosphate-solubilizing microorganisms achieve the dissolution of insoluble inorganic phosphate through secreting IAA to induce plant root system to produce acids, which has not been reported in relevant literature.

In recent years, increasing numbers of studies have used molecular biology methods to explore the mechanisms of phosphate solubilization by phosphate-solubilizing microorganisms. Babu-Khan and Kuhad cloned the phosphate-solubilizing genes in microorganisms [45]. Yuan studied phosphorus regulons, which were related to the dissolution of inorganic phosphorus in the* Sinorhizobium meliloti* genome [46, 47]. Meyer studied the glucose dehydrogenase that controls GA secretion in* Pseudomonas* and its cofactor pyrroloquinoline quinone [48]. In the present study,* Burkholderia multivorans* WS-FJ9 was shown to be an excellent and highly-effective phosphate-solubilizing strain. The molecular mechanisms of its phosphate solubilization as well as the cloning and expression of relevant phosphate-solubilizing genes require further exploration.

## 5. Conclusion

The bacterial strain of* Burkholderia multivorans* WS-FJ9 was found to have an excellent ability to solubilize inorganic phosphate with a significant effect on promoting poplar growth and was able to colonize the poplar rhizosphere. It not only can secrete organic acid by itself, but also can promote the root of poplar to secrete organic acid, especially the secretion of gluconic acid (GA),which in turn promotes the dissolution of inorganic phosphorus. After* B. multivorans* WS-FJ9 interacted with the poplar root system, the key phosphate-solubilizing driving force was gluconic acid (GA) which was produced in three ways: (1) secreted by the root system in the presence of IAA produced by* B. multivorans* WS-FJ9; (2) secreted by* B. multivorans* WS-FJ9; and (3) secreted by the poplar root system in the presence of phosphorus stress. When phosphorus stress was absent, the GA was produced as outlined in (1) and (2) above. These results demonstrated that inoculating* B. multivorans* WS-FJ9 into the poplar root system could increase the amount of GA secretion and implied that the interaction between* B. multivorans* WS-FJ9 and the poplar root system showed a strong capacity for solubilizing phosphate. We believe that these results are very valuable for boosting the study of the mechanism and pathways of solubilizing inorganic phosphate.

## Figures and Tables

**Table 1:** Experimental nutrient solution.

	0-P	Low-P	Normal-P	High-P
CaNO_3_·4H_2_O/mg	945	945	945	945
KNO_3_/mg	506	506	506	506
NH_4_NO_3_/mg	80	80	80	80
KH_2_PO_4_/mg	0	68	136	272
KCl/mg	74.6	37.3	0	0
Ferrous salt solution*∗*/mL	2.5	2.5	2.5	2.5
Trace element solution*∗∗*/mL	5	5	5	5
Water/mL	1000	1000	1000	1000
pH	6.8	6.7	6.6	6.4

*∗* Ferrous salt solution: 2.78 g of FeSO_4_·7H_2_O, 3.73 g of Na-EDTA, dissolved in 500 mL of distilled water.

*∗∗* Trace element solution: containing 0.83 mg·L^–1^ KI, 6.2 mg·L^–1^ H_3_BO_3_, 0.25 mg·L^–1^ MnSO_4_·H_2_O, 8.6 mg·L^–1^ ZnSO_4_, 0.25 mg·L^–1^ Na_2_MoO_4_·2H_2_O, 0.025 mg·L^–1^ CuSO_4_·5H_2_O, and 0.025 mg·L^–1^ CoCl_2_·6H_2_O.

**Table 2:** Concentration and pH of aqueous solution of exogenous organic acid.

	Gluconic acid	Shikimic acid	Lactic acid	Oxalic acid	Malonic acid
Concentration/mg·L^–1^	667.35	136.85	87.48	14.06	14.75
pH	6.3	6.6	6.7	6.8	6.7

**Table 3:** Tests of Between-Subjects Effects on the 2 × 4 × 2 factorial design experiment.

Source	Type III Sum of Squares	df	Mean Square	F	Sig.
Corrected Model	1.09E6^a^	15	72690.20	459.39	0.00
Intercept	860029.08	1	860029.08	5.44E3	0.00
A	512847.52	1	512847.52	3.24E3	0.00
B	119635.22	3	39878.41	252.02	0.00
C	287739.17	1	287739.17	1.82E3	0.00
A × B	26768.49	3	8922.83	56.39	0.00
A × C	112865.32	1	112865.32	713.28	0.00
B × C	30202.42	3	10067.47	63.62	0.00
A × B × C	294.81	3	98.27	0.62	0.61
Error	5063.48	32	158.23		
Total	1955445.50	48			
Corrected Total	1095416.42	47			

A: inoculation of *B. multivorans* WS-FJ9; B: phosphorus content of the nutrient solutions; C: poplar root system; a: R Squared =0.995 (adjusted R Squared =0.993); dependent variable: the amounts of GA production (mg·L^–1^).

**Table 4:** Multiple comparison of the 2 × 4 × 2 factorial design experiment.

A		B		C		A × B		A × C		B × C		A × B × C	
A1	237.22 a	B1	208.25 a	C1	211.28 a	A1 × B1	342.44 a	A1 × C1	363.14 a	B1 × C1	324.90 a	A1 × B1 × C1	503.44 a
A2	30.49 b	B2	148.63 b	C2	56.43 b	A1 × B2	264.96 b	A1 × C2	111.31 b	B2 × C1	230.33 b	A1 × B2 × C1	397.44 b
		B3	100.26 bc			A1 × B3	190.49 c	A2 × C1	59.42 c	B3 × C1	158.55 c	A1 × B3 × C1	298.65 c
		B4	78.28 c			A1 × B4	150.99 c	A2 × C2	1.56 d	B4 × C1	131.34 cd	A1 × B4 ×C1	253.01 d
						A2 × B1	74.05 d			B1 × C2	91.59 cde	A1 × B1 × C2	181.44 e
						A2 × B2	32.31 d			B2 × C2	66.94 de	A2 × B1 × C1	146.36 f
						A2 × B3	10.03 d			B3 × C2	41.97 e	A1 × B2 × C2	132.48 f
						A2 × B4	5.57 d			B4 × C2	25.22 e	A1 × B3 × C2	82.33 g
												A2 × B2 × C1	63.21 gh
												A1 × B4 × C2	48.97 h
												A2 × B3 × C1	18.45 i
												A2 × B4 × C1	9.68 i
												A2 × B1 × C2	1.75 i
												A2 × B3 × C2	1.61 i
												A2 × B4 × C2	1.47 i
												A2 × B2 × C2	1.40 i

A: inoculation of *B. multivorans* WS-FJ9 (A1: inoculated with WS-FJ9; A2: not inoculated with WS-FJ9); B: content of phosphorus (B1: 0-phosphorus; B2: low-phosphorus; B3: normal-phosphorus; B4: high-phosphorus); C: poplar root system (C1: planted with poplar; C2: not planted with poplar); dependent variable: the amounts of GA production (mg·L^–1^).

## Data Availability

The data used to support the findings of this study are available from the corresponding author upon request.
